# Effect of intermittent theta burst stimulation of the left DLPFC on cognitive function and inflammatory markers in post-stroke cognitive impairment: a randomized controlled trial

**DOI:** 10.3389/fpsyt.2025.1686265

**Published:** 2025-11-05

**Authors:** Jiayi Xia, Yeping Chen, Xiaoyan Jiang, Song Pei

**Affiliations:** ^1^ Clinical Research Center, The Second Rehabilitation Hospital of Shanghai, Shanghai, China; ^2^ Department of Rehabilitation Medicine, Renhe Hospital, Baoshan District, Shanghai, China

**Keywords:** post-stroke cognitive impairment (PSCI), intermittent theta- burst stimulation(iTBS), dorsolateral prefrontal cortex (DLPFC), cognitive rehabilitation, inflammatorybiomarker

## Abstract

**Objective:**

To evaluate the efficacy of intermittent theta-burst stimulation (iTBS) targeting the left dorsolateral prefrontal cortex (DLPFC) combined with cognitive training in patients with post-stroke cognitive impairment (PSCI), and to investigate its effects on systemic inflammatory biomarkers: homocysteine (Hcy), C-reactive protein (CRP), and lactate dehydrogenase (LDH).

**Methods:**

In this single-center, randomized, sham-controlled trial, 69 PSCI patients received 4 weeks of daily cognitive training combined with either real iTBS (target: left DLPFC; 1200 pulses per session at 80 % of resting motor threshold, total 20 sessions) or sham stimulation. Assessments were conducted at baseline (week 0) and week 4, including measures of global cognition (Mini-Mental State Examination [MMSE], Montreal Cognitive Assessment [MoCA]), executive function (Frontal Assessment Battery [FAB]), activities of daily living (Barthel Index [BI]), domain-specific cognitive subscores (forward/backward digit span [FDS/BDS], delayed recall, attention), and inflammatory biomarkers (Hcy, CRP, LDH). Data were analyzed using two-way mixed General Linear Models (GLM) to assess main and interaction effects of Time and Group.

**Results:**

Significant Time effects were observed for all cognitive and biochemical measures (p < 0.001), indicating overall improvement after intervention. Significant Time × Group interactions favored the iTBS group for MMSE, MoCA, BI, FDS, BDS (p < 0.05), suggesting enhanced gains in global cognition, executive function, and working memory. Serum LDH showed a greater reduction in the iTBS group (p < 0.05), while decreases in Hcy and CRP were comparable between groups. Correlation analysis revealed that reductions in LDH and Hcy were significantly associated with improvements in MMSE, MoCA, FAB, and working-memory subscores in the iTBS group (r = −0.334 to −0.525, p < 0.05), supporting a metabolic-cognitive coupling effect.

**Conclusions:**

iTBS applied to the left DLPFC, combined with cognitive training, produces superior improvements in global cognition, executive function, and daily living ability compared with cognitive training alone in PSCI patients. The concomitant reduction in LDH suggests potential anti-inflammatory or neuroprotective mechanisms underlying these cognitive benefits. LDH may thus serve as a sensitive peripheral biomarker for neuromodulation-induced recovery in PSCI rehabilitation.

**Clinical trial registration:**

https://www.chictr.org.cn/, identifier ChiCTR2300076109.

## 1 Introduction

Post-stroke cognitive impairment (PSCI) is one of the most debilitating sequelae of stroke, affecting up to 60% of survivors within the first year and persisting in roughly one-third long-term (1). Contemporary epidemiological syntheses suggest an overall prevalence as high as 70%, making PSCI a leading contributor to post-stroke disability, dependency and rehospitalisation (2). In addition to lowering quality of life, PSCI markedly increases health-care costs and hampers the recovery of motor, language and psychosocial functions, thereby amplifying the overall burden on families and society (3).

Despite three decades of research, the mechanisms underlying PSCI remain incompletely understood. Growing evidence points to a pivotal contribution of systemic and cerebral inflammation. Large prospective cohorts have shown that elevated circulating C-reactive protein (CRP), homocysteine (Hcy) and lactate dehydrogenase (LDH) soon after stroke predict early-onset cognitive decline (4). Meta-analytic work further implicates CRP, Hcy, total cholesterol and LDL-C as independent biomarkers of PSCI risk (5). Inflammatory panels derived from high-throughput proteomics now explain up to 35% of variance in cognitive outcomes at 12 months (6), highlighting neuro-immune interactions as a therapeutic target.

Pharmacological options for PSCI are limited and largely off-label, underscoring the need for disease-modifying strategies. Repetitive transcranial magnetic stimulation (rTMS) is a non-invasive brain-stimulation technique that modulates cortical excitability and has demonstrated moderate efficacy in multiple systematic reviews (7). A 2024 network meta-analysis comparing rTMS protocols ranked theta-burst paradigms among the most promising but highlighted heterogeneity and small sample sizes (8).

iTBS delivers bursts of 50 Hz pulses repeated at the endogenous 5 Hz theta rhythm, requiring fewer pulses, lower intensities and < 5 min per session while producing longer-lasting neuroplastic effects than conventional high-frequency rTMS. A single-blind randomized controlled trial in 2023 showed that iTBS over the left dorsolateral prefrontal cortex (DLPFC) combined with cognitive training significantly improved MoCA and executive scores versus sham in PSCI (9). High-dose, individualized iTBS protocols further enhanced global cognition without compromising safety (10), and adjunctive approaches such as scalp-acupuncture-primed iTBS yielded additive benefits (11). Bibliometric analyses confirm iTBS as a rapidly expanding research hotspot in neuromodulation (12). Beyond cognition, iTBS accelerates motor recovery and network re-organization in early stroke (13), promotes neurovascular unit remodeling (14), and mitigates ferroptotic and apoptotic cascades after ischaemia-reperfusion injury (15), underscoring its pleiotropic potential.

While mechanistic reviews suggest that theta-burst stimulation down-regulates oxidative stress, glial activation and pro-inflammatory cytokine release (16), direct clinical evidence linking iTBS-induced cognitive gains to changes in peripheral or central inflammatory markers is scarce. Pre-clinical studies demonstrate that iTBS shifts microglia toward an M2 reparative phenotype via Cry1 signaling (17) and enhances PI3K/Akt-mediated synaptic plasticity while dampening neuro-inflammation (18). Meta-analytic data on theta-burst stimulation for motor recovery likewise imply anti-inflammatory actions (19), yet clinical correlation with biomarkers such as LDH or CRP in PSCI is virtually unexplored. Continuous-TBS paradigms can even exert opposite dopaminergic and excitability effects, highlighting the need for protocol-specific biomarker mapping (20).

Given (i) the high prevalence and societal impact of PSCI, (ii) compelling but inconclusive evidence for iTBS-mediated cognitive restoration, and (iii) the putative role of systemic inflammation in cognitive decline, we designed a randomized controlled trial to determine whether intermittent theta-burst stimulation of the left DLPFC enhances cognitive recovery in PSCI and whether such effects are accompanied by favorable shifts in inflammatory biomarkers (Hcy, CRP, LDH). Clarifying these relationships will help refine precision-neuromodulation strategies and provide mechanistic insight into non-pharmacological treatment of PSCI.

## 2 Methods

### 2.1 Subjects

**Figure 1:**
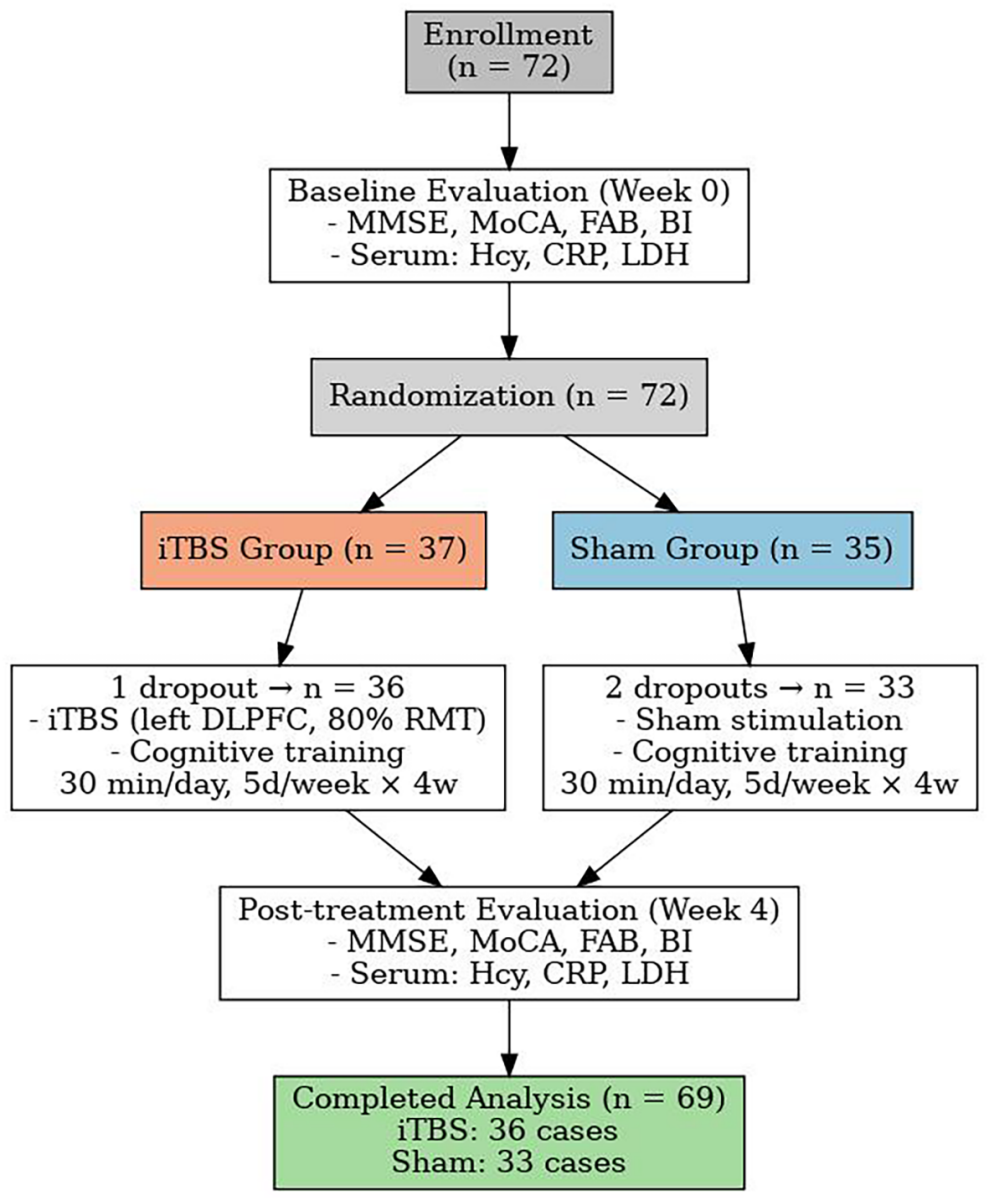
The flow-chart of the study.

This single-center, parallel-group, assessor-blinded, randomized, sham-controlled trial was conducted in the Second Rehabilitation Hospital of Shanghai between 1 January 2023 and 31 December 2024. Trial reporting follows the CONSORT-2010 extension for non-pharmacological interventions (21) and adopts the updated IFCN safety recommendations for transcranial magnetic stimulation (TMS) (22). A total of 72 eligible patients were enrolled in the study. During the intervention period, 3 patients withdrew due to early discharge from the hospital—1 from the iTBS group and 2 from the sham group. Ultimately, 69 participants completed the study, including 36 in the iTBS group and 33 in the sham group (see **Figure 1**).

#### 2.1.1 Eligibility criteria

Inclusion criteria were:

- Diagnosis of PSCI according to the 2019 Chinese Expert Consensus on post-stroke cognitive impairment (23) and harmonized with the NINDS-CSN vascular cognitive impairment standards (24).
- First-ever ischaemic or haemorrhagic stroke confirmed by CT or MRI within 6 months (25).
- Age 45–80 years.
- Mini-Mental State Examination (MMSE) ≤ 24 at screening, indicating at least mild cognitive deficit.
- Stable medical condition and ability to participate in cognitive testing and rehabilitation.
- Provision of written informed consent by the patient or legally authorized representative in accordance with the Declaration of Helsinki.

Exclusion criteria were:

- Contra-indications to TMS (metallic cranial implants, cardiac pacemaker, active epilepsy, skull defects) per IFCN guidelines (26).
- Pre-existing neurodegenerative or severe psychiatric disorders.
- Severe aphasia, visual or auditory deficits precluding valid cognitive assessment.
- Uncontrolled systemic disease (eg, de-compensated heart failure, severe renal/hepatic insufficiency).
- Current participation in another interventional trial.

#### 2.1.2 Sample-size determination

*A priori* power analysis was performed with G*Power 3.1.9 using the difference in Montreal Cognitive Assessment (MoCA) improvement reported in a recent iTBS-PSCI pilot (mean ± SD change 3.2 ± 4.0 vs 0.6 ± 3.5) (9). Assuming a two-tailed α = 0.05, 80% power and an effect size d = 0.7, we required 33 participants per arm. Anticipating a 15% attrition rate, we set the target enrolment at 78 subjects (39 each group), consistent with recommendations for pragmatic clinical trials (5).

During the recruitment period, 72 eligible patients meeting all inclusion criteria were successfully enrolled, and 69 participants completed the full intervention and assessment schedule (iTBS = 36, Sham = 33). The actual attrition rate of ≈ 8% was lower than anticipated, and *post hoc* power analysis confirmed that the achieved statistical power remained above 0.80 for detecting the observed group × time interaction effects in the General Linear Model (GLM) analysis.

#### 2.1.3 Randomization and masking

Participants were randomly assigned (1: 1) to either intermittent theta-burst stimulation (iTBS) or sham stimulation using a computer-generated sequence with random block sizes of 4–6 prepared by an independent statistician. Allocation was concealed in sequentially numbered, opaque, sealed envelopes opened by the TMS operator immediately before the first session. Outcome assessors, data analysts and participants were blinded to group allocation. Sham stimulation was delivered with the figure-of-”8” coil rotated 90° away from the scalp, reproducing acoustic artefacts without effective cortical stimulation (6).

#### 2.1.4 Baseline assessment

Demographic data (age, sex, education), stroke characteristics (type) and cognitive/functional status (Mini-Mental State Examination (MMSE), Montreal Cognitive Assessment (MoCA), Barthel Index (BI)) were collected at enrolment. Resting motor threshold was measured over the contralesional “motor hotspot” according to standardized procedures (27). Peripheral blood was drawn for inflammatory biomarkers (homocysteine (Hcy), C-reactive protein (CRP), Lactate dehydrogenase (LDH)) before randomization, aligning with emerging evidence linking these markers to PSCI severity (28). The demographic characteristics and baseline MMSE scores did not differ significantly between the two groups (P > 0.05; see **Table 1**).

**Table 1:** Baseline demographic and clinical characteristics of participants in the iTBS and sham-stimulation groups.

Variable	iTBS group (n=36)	Sham group (n=33)	Test statistic (χ^2^/t/Z)	*P-value*
Male/Female (n/n)	21/15	25/8	2.352	0.125
Stroke type			0.643	0.423
ischaemic	31	26		
haemorrhagic	5	7		
Age (mean ± SD, years)	64.39 ± 5.52	66.79 ± 6.74	-1.624	0.109
Duration since stroke (months); median (Q1, Q3)	3.00(2.00,4.00)	2.00(2.00,4.00)	-1.179	0.238
Years of education (years); median (Q1, Q3)	9.00(6.00,12.00)	6.00(6.00,9.00)	-1.902	0.057
MMSE score; median (Q1, Q3)	16.00(6.25,23.00)	15.00(9.00,20.50)	-0.548	0.584

Itbs, intermittent theta-burst stimulation; MMSE, Mini-Mental State Examination.

#### 2.1.5 Ethics and registration

The study protocol was approved by the Shanghai Second Rehabilitation Hospital Ethics Committee (approval No. 2022-10-01) and registered at the Chinese Clinical Trial Registry (ChiCTR2300076109). All procedures complied with the Declaration of Helsinki and local regulatory requirements.

### 2.2 Evaluation indicators

We adopted a multimodal evaluation battery that captures global cognition, frontal-executive control, functional independence, working-memory span and systemic inflammation. All tools have been validated in stroke cohorts during the past five years and align with contemporary PSCI-assessment recommendations.

#### 2.2.1 Global cognition

MMSE (0–30) and MoCA (0–30; +1 point if ≤12 y education) were administered. A 2024 multicenter analysis confirmed comparable discrimination of MMSE and MoCA for PSCI detection, supporting continued use of both screeners (29). However, an updated diagnostic-accuracy study recommends a stroke-specific MoCA cut-off of 21/22 to optimize sensitivity and specificity in Asian populations (30).

#### 2.2.2 Frontal-executive function

FAB (0–18) probes abstract reasoning, mental set-shifting and inhibitory control at the DLPFC level. A 2024 Japanese validation showed high internal consistency (α = 0.89) and limits of agreement of −1.7 to +2.9 points in stroke survivors (31); similar reliability was reported for the telephone FAB variant in 2022 (32).

#### 2.2.3 Activities of daily living

Functional independence was quantified with the BI (0–100). Machine-learning prognostic modelling (2024) demonstrated that baseline BI scores accurately predict discharge self-care status after intensive stroke rehabilitation (33), while item-level analyses in a 2023 registry identified grooming and transfers as the strongest early predictors of global BI at discharge (34).

#### 2.2.4 Working-memory span

Forward Digit-Span (FDS) and Backward Digit-Span (BDS) were delivered according to WAIS-IV procedures. A decade-long longitudinal study revealed that BDS trajectories closely mirror functional-connectivity changes and long-term cognitive recovery post-stroke (35). Complementary evidence from a 2023 cardiovascular-risk cohort linked BDS decline to vascular-cognitive trajectories in 137 stroke survivors (36).

#### 2.2.5 Delayed recall

We assessed delayed recall using the MoCA’s memory subscore (range 0–5). This subitem—a 5-word recall following a 5-minute delay—is sensitive to hippocampal-dependent consolidation deficits common in post-stroke cognitive impairment. Domain-specific analyses have demonstrated that delayed recall declines significantly between discharge and 3-month follow-up post-stroke, highlighting its longitudinal sensitivity and prognostic value for functional outcomes (37).

#### 2.2.6 Attention

The attention domain of MoCA (range 0–6) includes tasks assessing sustained attention, working memory (digit span), and serial subtraction. Recent studies emphasize its robust association with executive control network dysfunction and daily activity performance post-stroke. Prognostic modeling studies have confirmed that attention subscores contribute valuable specificity in detecting PSCI, particularly when combined with other domain scores (38, 39).

#### 2.2.7 Inflammatory & metabolic biomarkers

Fasting venous samples (07:00–08:00 h) were collected for: Hcy (μmol/L); CRP (mg/L); LDH (U/L). A 2023 meta-analysis involving > 3–000 patients confirmed that elevated Hcy and CRP independently predict early cognitive decline after acute stroke (40, 41). Two large observational studies reported that high LDH or an elevated LDH-to-albumin ratio is associated with poor 3-month neurological outcome and larger infarct burden (42, 43).

All evaluation indicators—MOCA total, MMSE, FAB, BI, FDS/BDS, delayed recall, attention subscores, and inflammatory biomarkers—were measured at baseline (week 0) and repeated at end of week 4, ensuring consistency in pre- and post-treatment comparisons.

### 2.3 Intervention protocol

#### 2.3.1 Overview of intervention

Both study arms received standard-of-care baseline treatment—comprising conventional pharmacotherapy (e.g. antiplatelets, statins, antihypertensives) and individualized rehabilitation (physiotherapy, occupational therapy, physical modalities)—alongside structured one-on-one cognitive training lasting 30 minutes daily. Cognitive tasks focused on everyday functional relevance and engagement, including object recognition/use, memory card tasks, numeracy tasks, and computer-assisted training, with graduated difficulty tailored to patient ability. After cognitive training, participants proceeded to group-specific experimental treatments. Sessions occurred once daily, five times per week for four consecutive weeks (total 20 sessions). Outcomes were assessed at week 0 and week 4 endpoints.

#### 2.3.2 iTBS treatment (experimental group)

**Figure 2:**
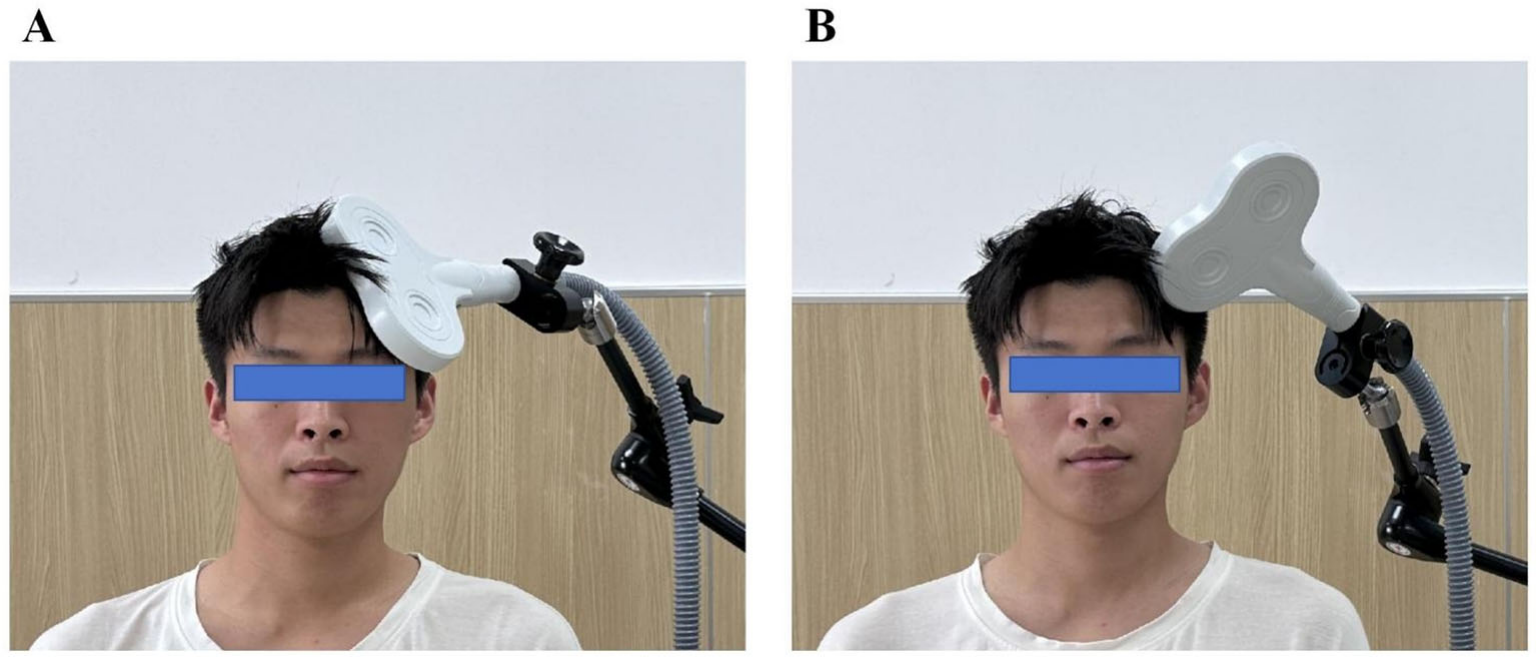
Stimulation Protocols for the iTBS and Sham Groups. **(A)** Real intermittent theta burst stimulation (iTBS) in the iTBS group: the figure-of-”8” coil was positioned tangentially to the scalp over the left dorsolateral prefrontal cortex (DLPFC) at an angle that ensured effective cortical stimulation. **(B)** Sham stimulation in the control group: the coil was rotated 90° perpendicular to the scalp surface, minimizing magnetic field penetration and producing only auditory and somatosensory sensations without real cortical activation.

The experimental group received intermittent theta-burst stimulation (iTBS) delivered with a OSF-5/T TMS device (Aosaifu, Wuhan, China) using a figure-of-“8” coil. The iTBS protocol consisted of bursts of three pulses at 50 Hz, repeated at 5 Hz (theta rhythm). Specifically, a 2 s stimulation train was followed by an 8 s pause, repeated until a total of 1200 pulses over approximately 383.7 s (~6.4 min) per session. Stimulation intensity was set at 80 % of the resting motor threshold (RMT) measured at the contralesional M1 “motor hotspot” in sitting subjects with relaxed upper limb muscles, defined as the minimal intensity producing ≥50 µV MEP in 5 of 10 trials. The coil was placed tangentially over the left dorsolateral prefrontal cortex (DLPFC)—identified with standard anatomical landmarks and the international 10—20 System—consistent with recent clinical protocols for PSCI rehabilitation (see **Figure 2A**).

#### 2.3.3 Sham stimulation (control group)

Sham treatment used an identical schedule and device settings, but with the coil rotated 90°, producing similar acoustic and sensory artifacts without effective cortical stimulation. All other parameters (intensity, duration, coil type, session frequency) were matched to the active iTBS group. Participants were instructed to remain relaxed and stable throughout treatment, and the coil position was consistently marked to ensure precision over the four-week course (see **Figure 2B**).

#### 2.3.4 Safety monitoring and stimulation precision

RMT was re-assessed weekly to accommodate potential fluctuations in cortical excitability and to adjust stimulation dosage accordingly. To promote safety, operators monitored for adverse events (e.g. headache, scalp discomfort, syncope) after each session, in line with updated NIBS safety guidelines (no serious adverse effects were reported in prior iTBS-PSCI trials) (44, 45). Coil positioning and patient head stability were verified before each session to maintain consistent targeting across treatment days.

### 2.4 Statistical analysis

All statistical analyses were performed using IBM SPSS Statistics 26.0 (IBM Corp., Armonk, NY, USA). Data distribution was examined using the Shapiro–Wilk test. Continuous variables conforming to a normal distribution are presented as the mean ± standard deviation (SD), while non-normally distributed data are expressed as the median (interquartile range, IQR). Categorical variables are summarized as counts and percentages.

Between-group differences in baseline demographic and clinical characteristics (**Table 1**) were analyzed using the independent-samples t test for normally distributed variables, the Mann–Whitney U test for non-normal variables, and the chi-square (χ²) test for categorical data.

To evaluate treatment effects, cognitive, subdomain, and biochemical outcomes were analyzed using a two-way mixed-design General Linear Model (GLM) with Group (iTBS vs. Sham) as the between-subjects factor and Time (Pre vs. Post) as the within-subjects factor (**Table 2**). The interaction term (Time × Group) reflected the differential effect of iTBS relative to sham stimulation. Where significant main or interaction effects were detected, Bonferroni-corrected *post hoc* tests were performed. Partial η² values were calculated to estimate effect size and interpreted as small (≥ 0.01), medium (≥ 0.06), and large (≥ 0.14).

**Table 2:** General Linear Model (GLM) results for cognitive, subdomain, and biochemical outcomes in the iTBS and Sham groups.

Outcome measure	Source of variation	F	df	P-value	Partial η²	Interpretation
MMSE	Time (Pre vs. Post)	39.001	1,67	<0.001	0.368	Significant Time effect; overall cognition improved after treatment.
	Group (iTBS vs. Sham)	3.318	1,67	0.073	0.047	Trend toward higher overall scores in iTBS.
	Time × Group	9.26	1,67	0.003	0.121	iTBS group showed greater MMSE improvement than Sham.
MoCA	Time (Pre vs. Post)	97.569	1,67	<0.001	0.593	Strong Time effect; marked global cognitive improvement.
	Group (iTBS vs. Sham)	4.458	1,67	0.038	0.062	Overall higher MoCA scores in iTBS group.
	Time × Group	4.503	1,67	0.038	0.063	Greater cognitive gain in iTBS vs. Sham.
FAB	Time (Pre vs. Post)	74.914	1,67	<0.001	0.528	Executive function improved significantly in both groups.
	Group (iTBS vs. Sham)	7.207	1,67	0.009	0.097	Higher overall FAB scores in iTBS.
	Time × Group	2.3	1,67	0.134	0.033	No significant interaction; both groups improved similarly.
BI	Time (Pre vs. Post)	74.63	1,67	<0.001	0.527	Activities of daily living improved after therapy.
	Group (iTBS vs. Sham)	3.493	1,67	0.066	0.05	Trend toward higher overall BI in iTBS.
	Time × Group	7.801	1,67	0.007	0.104	iTBS yielded greater functional gains than Sham.
FDS	Time (Pre vs. Post)	45.103	1,67	<0.001	0.402	Working-memory span increased after treatment.
	Group (iTBS vs. Sham)	3.476	1,67	0.067	0.049	Trend favoring iTBS overall.
	Time × Group	5.281	1,67	0.025	0.073	Stronger FDS improvement in iTBS.
BDS	Time (Pre vs. Post)	35.413	1,67	<0.001	0.346	Backward-digit memory improved across time.
	Group (iTBS vs. Sham)	8.724	1,67	0.004	0.115	Higher overall BDS in iTBS group.
	Time × Group	14.827	1,67	<0.001	0.181	Marked interaction: iTBS produced greater improvement.
Delayed Recall	Time (Pre vs. Post)	49.644	1,67	<0.001	0.426	Memory recall improved after treatment.
	Group (iTBS vs. Sham)	0.847	1,67	0.361	0.012	No group difference.
	Time × Group	0.209	1,67	0.649	0.003	No differential effect between groups.
Attention	Time (Pre vs. Post)	34.135	1,67	<0.001	0.338	Attention enhanced over time.
	Group (iTBS vs. Sham)	3.521	1,67	0.065	0.05	Trend toward better performance in iTBS.
	Time × Group	2.953	1,67	0.09	0.042	No significant interaction; both groups improved.
Hcy (μmol/L)	Time (Pre vs. Post)	26.653	1,67	<0.001	0.285	Homocysteine decreased post-treatment in both groups.
	Group (iTBS vs. Sham)	0.086	1,67	0.770	0.001	No group difference.
	Time × Group	0.429	1,67	0.515	0.006	No interaction effect.
CRP (mg/L)	Time (Pre vs. Post)	15.354	1,67	<0.001	0.186	Inflammatory marker decreased after treatment.
	Group (iTBS vs. Sham)	0.029	1,67	0.865	<0.001	No difference between groups.
	Time × Group	0.178	1,67	0.675	0.003	No interaction effect.
LDH (U/L)	Time (Pre vs. Post)	33.603	1,67	<0.001	0.334	LDH decreased significantly after intervention.
	Group (iTBS vs. Sham)	3.172	1,67	0.079	0.045	Trend toward lower LDH in iTBS.
	Time × Group	2.899	1,67	0.093	0.041	Slight trend for stronger LDH reduction in iTBS.

Results are derived from a two-way mixed General Linear Model (GLM) with factors Time (Pre vs Post) and Group (iTBS vs Sham). F values, degrees of freedom (df), p-values, and partial η² (effect sizes) are reported. Partial η² values are interpreted as small (≥ 0.01), medium (≥ 0.06), and large (≥ 0.14). p < 0.05 was considered statistically significant.

iTBS, intermittent theta-burst stimulation; MMSE, Mini-Mental State Examination; MoCA, Montreal Cognitive Assessment; FAB, Frontal Assessment Battery; BI, Barthel Index; FDS, forward digit span; BDS, backward digit span; Hcy, homocysteine; CRP, C-reactive protein; LDH, lactate dehydrogenase.

For descriptive reference, paired-samples t-tests were used within each group to identify significant pre-to-post changes, and independent-samples t-tests were used to compare post-treatment values between the iTBS and sham groups (**Table 3**).

**Table 3:** Descriptive statistics for cognitive, subdomain, and biochemical outcomes (Mean ± SD).

Outcome measure	Group	n	Pre	Post	Δ Change	T-value	*P-value*
MMSE	iTBS	36	15.08 ± 8.28	21.50 ± 6.93^**^	6.42 ± 6.95	5.543	<0.001^*^
	Sham	33	14.30 ± 6.89	16.52 ± 6.32	2.21 ± 4.01	3.172	0.003^*^
MoCA	iTBS	36	11.44 ± 6.73	18.81 ± 6.46^**^	7.36 ± 5.93	7.443	<0.001^*^
	Sham	33	9.73 ± 6.00	14.48 ± 6.59	4.76 ± 3.97	6.886	<0.001^*^
FAB	iTBS	36	8.25 ± 4.49	12.61 ± 4.28^**^	4.36 ± 4.27	6.127	<0.001^*^
	Sham	33	6.48 ± 3.73	9.55 ± 3.95	3.06 ± 2.56	6.865	<0.001^*^
BI	iTBS	36	45.56 ± 24.95	67.78 ± 22.85^**^	22.22 ± 17.58	7.583	<0.001^*^
	Sham	33	41.97 ± 19.92	53.33 ± 17.35	11.36 ± 14.38	4.541	<0.001^*^
FDS	iTBS	36	4.61 ± 2.36	6.53 ± 2.46^**^	1.92 ± 2.29	5.033	<0.001^*^
	Sham	33	4.21 ± 1.82	5.15 ± 1.91	0.94 ± 0.90	6.001	<0.001^*^
BDS	iTBS	36	1.97 ± 1.23	3.53 ± 1.50^**^	1.56 ± 1.61	5.792	<0.001^*^
	Sham	33	1.70 ± 1.49	2.03 ± 1.40	0.33 ± 0.89	2.152	0.039^*^
Delayed Recall	iTBS	36	0.78 ± 0.99	1.92 ± 1.42	1.14 ± 1.44	4.754	<0.001^*^
	Sham	33	0.64 ± 0.90	1.64 ± 1.17	1.00 ± 1.03	5.573	<0.001^*^
Attention	iTBS	36	2.67 ± 2.07	4.11 ± 1.92^**^	1.44 ± 1.80	4.826	<0.001^*^
	Sham	33	2.18 ± 1.90	2.97 ± 1.96	0.79 ± 1.32	3.436	0.002^*^
Hcy (μmol/L)	iTBS	36	13.542 ± 4.08	10.22 ± 3.42	-3.32 ± 4.65	4.285	<0.001^*^
	Sham	33	13.382 ± 4.91	10.81 ± 2.61	-2.57 ± 4.83	3.060	0.004^*^
CRP (mg/L)	iTBS	36	7.15 ± 11.19	3.77 ± 8.10	-3.37 ± 6.49	3.116	0.004^*^
	Sham	33	6.45 ± 11.20	3.73 ± 6.51	-2.72 ± 6.40	2.440	0.020^*^
LDH (U/L)	iTBS	36	158.69 ± 37.01	126.28 ± 18.01^**^	-32.42 ± 34.96	5.563	<0.001^*^
	Sham	33	164.39 ± 42.95	146.70 ± 39.06	-17.70 ± 36.84	2.760	0.009^*^

*Indicates a statistically significant difference within the same group (before vs. after treatment) (P<0.05). **Indicates a statistically significant difference between groups (iTBS vs. sham) after treatment (P<0.05). Values are expressed as mean ± standard deviation (SD). Δ Change = Post – Pre. p < 0.05 was considered statistically significant. iTBS, intermittent theta-burst stimulation; MMSE, Mini-Mental State Examination; MoCA, Montreal Cognitive Assessment; FAB, Frontal Assessment Battery; BI, Barthel Index; FDS, forward digit span; BDS, backward digit span; Hcy, homocysteine; CRP, C-reactive protein; LDH, lactate dehydrogenase.

**Figure 3:**
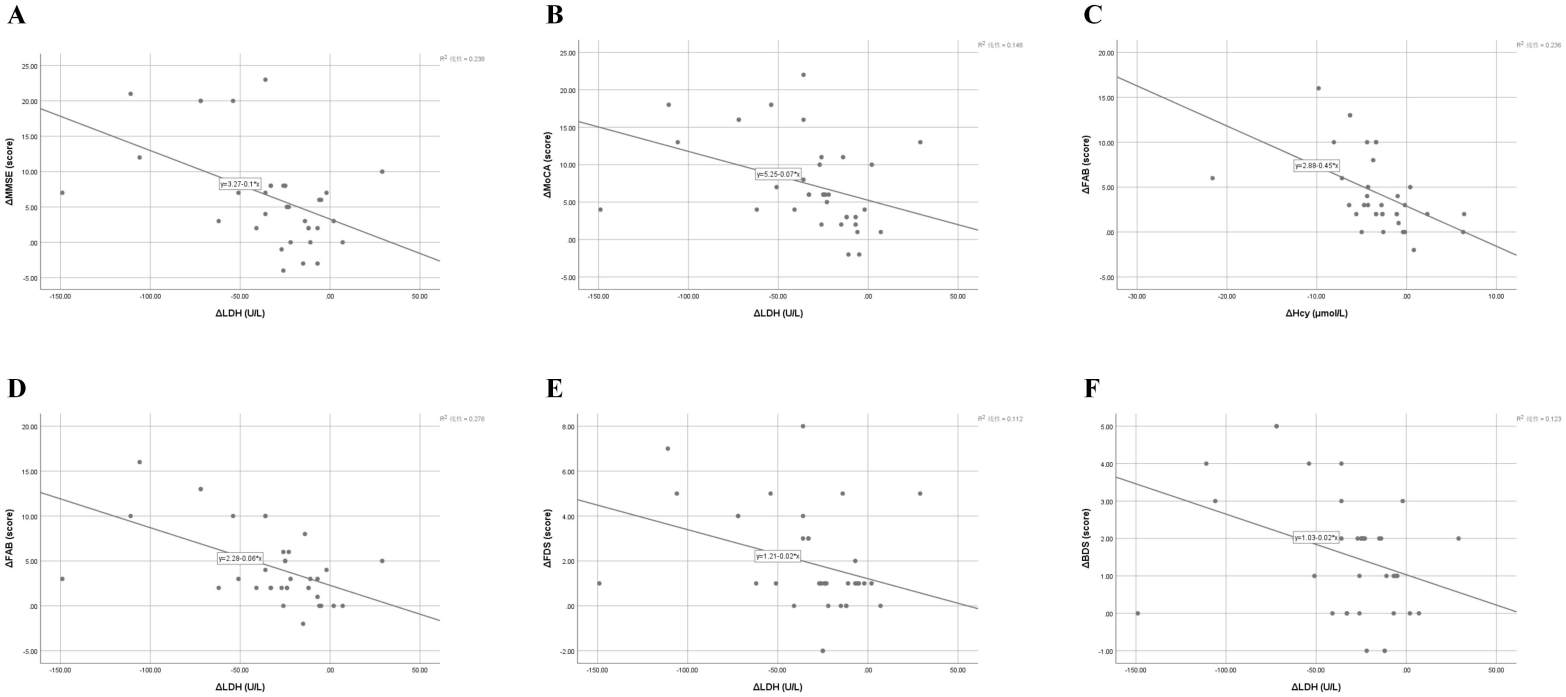
Correlations between changes in cognitive performance and biochemical markers following iTBS treatment. **(A)** Correlation between changes in Mini-Mental State Examination (ΔMMSE) and lactate dehydrogenase (ΔLDH) levels (r = –0.488, p = 0.003). **(B)** Correlation between changes in Montreal Cognitive Assessment (ΔMoCA) and ΔLDH levels (r = –0.384, p = 0.021). **(C)** Correlation between changes in Frontal Assessment Battery (ΔFAB) and homocysteine (ΔHcy) levels (r = –0.486, p = 0.003). **(D)** Correlation between changes in ΔFAB and ΔLDH levels (r = –0.525, p = 0.001). **(E)** Correlation between changes in forward digit span (ΔFDS) and ΔLDH levels (r = –0.334, p = 0.046). **(F)** Correlation between changes in backward digit span (ΔBDS) and ΔLDH levels (r = –0.351, p = 0.036). Each panel illustrates a significant or near-significant relationship between cognitive improvement and biochemical modulation after intermittent theta-burst stimulation (iTBS). Pearson’s correlation analysis revealed that greater reductions in serum LDH and Hcy were associated with larger gains in global cognition, executive function, and working memory. Solid lines represent the best-fit linear regression. r and p values are based on Pearson’s correlation analysis; mean ± SD values are used for descriptive reference. iTBS, intermittent theta-burst stimulation; MMSE, Mini-Mental State Examination; MoCA, Montreal Cognitive Assessment; FAB, Frontal Assessment Battery; FDS, forward digit span; BDS, backward digit span; Hcy, homocysteine; LDH, lactatedehydrogenase.

Correlations between changes in cognitive performance and biochemical parameters (**Table 4**; **Figure 3**) were examined using Pearson’s correlation analysis within each group. Δ values were computed as the difference between post- and pre-treatment scores (Δ = Post – Pre).

**Table 4:** Correlations between Changes in Cognitive and Biochemical Parameters after Treatment in the iTBS and Sham Groups.

Variable pair	Group	r	P-value
ΔMMSE – ΔLDH	iTBS	-0.488	0.003
ΔMoCA – ΔLDH	iTBS	-0.384	0.021
ΔFAB – ΔHcy	iTBS	-0.486	0.003
ΔFAB – ΔLDH	iTBS	-0.525	0.001
ΔFDS – ΔLDH	iTBS	-0.334	0.046
ΔBDS – ΔLDH	iTBS	-0.351	0.036
ΔMMSE – ΔHcy	Sham	0.687	<0.001
ΔMoCA – ΔHcy	Sham	0.469	0.006
ΔFDS – ΔHcy	Sham	0.382	0.028
ΔAttention – ΔHcy	Sham	0.607	<0.001

Values represent Pearson correlation coefficients (r) and corresponding two-tailed p-values. Δ indicates change from post- to pre-treatment (Δ = Post – Pre). p < 0.05 was considered statistically significant. iTBS, intermittent theta-burst stimulation; MMSE, Mini-Mental State Examination; MoCA, Montreal Cognitive Assessment; FAB, Frontal Assessment Battery; BI, Barthel Index; FDS, forward digit span; BDS, backward digit span; Hcy, homocysteine; CRP, C-reactive protein; LDH, lactate dehydrogenase.

All statistical tests were two-tailed, and a p value < 0.05 was considered statistically significant.

## 3 Results

### 3.1 Baseline characteristics

As shown in **Table 1**, no significant differences were found between the iTBS and sham groups in sex distribution, stroke type, age, time since stroke onset, years of education, or baseline MMSE scores (all p > 0.05). These results indicate good baseline comparability between the two groups prior to intervention.

### 3.2 Changes in global cognition and daily function

**Figure 4:**
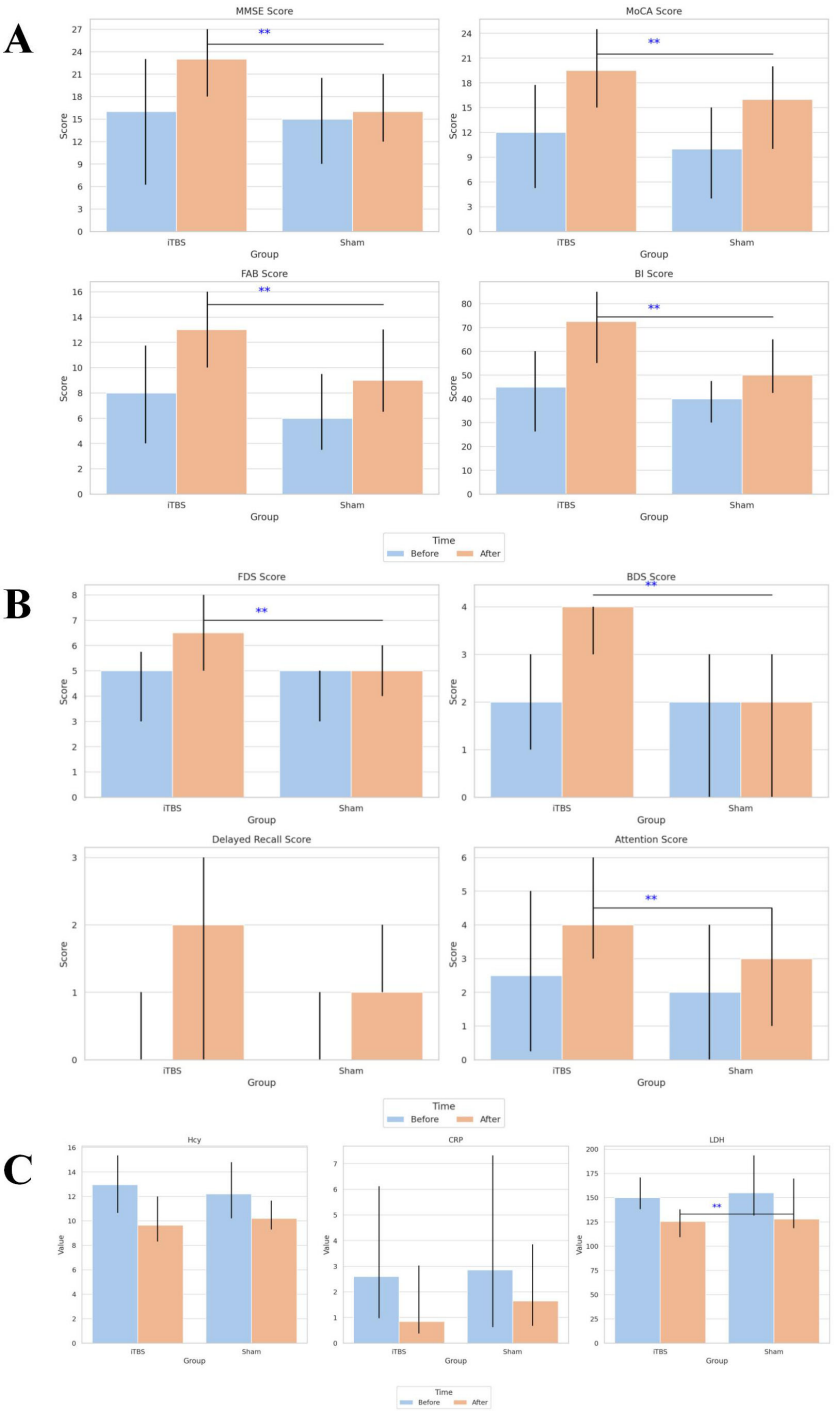
Comparison of cognitive, memory, and biochemical outcomes before and after treatment in the iTBS and sham groups. **(A)** Comparison of cognitive function and activities of daily living scores before and after treatment in the two groups. **(B)** Comparison of memory and attention scores before and after treatment in the two groups. **(C)** Comparison of serum biochemical indicators before and after treatment in the two groups. Values are presented as mean ± SD. **Indicates a statistically significant difference between groups (P < 0.05). iTBS, intermittent theta-burst stimulation; MMSE, Mini-Mental State Examination; MoCA, Montreal Cognitive Assessment; FAB, Frontal Assessment Battery; BI, Barthel Index; FDS, forward digit span; BDS, backward digit span; Hcy, homocysteine; CRP, C-reactive protein; LDH, lactatedehydrogenase.

After the 4-week intervention, both groups demonstrated significant within-group improvements in MMSE, MoCA, FAB, and BI scores compared with baseline (all p < 0.001). However, post-treatment comparisons revealed that the iTBS group achieved significantly greater gains than the sham group across all four scales (**Table 3**, **Figure 4A**).

The two-way mixed General Linear Model (GLM) confirmed significant main effects of Time for all cognitive and functional measures (all p < 0.001), indicating overall improvement after treatment. Moreover, significant Time × Group interactions for MMSE (F = 9.26, p = 0.003, partial η² = 0.121), MoCA (F = 4.503, p = 0.038, partial η² = 0.063), and BI (F = 7.801, p = 0.007, partial η² = 0.104) suggest that iTBS combined with cognitive training led to greater cognitive and functional improvements than sham stimulation (**Table 2**).

### 3.3 Changes in memory and attention subdomains

Baseline performance on the FDS, BDS, delayed recall, and attention subscores did not differ significantly between groups (all p > 0.05). Following intervention, both groups exhibited significant within-group improvements in all subdomains (all p < 0.05).

Between-group comparisons indicated that the iTBS group showed significantly greater improvements in FDS, BDS, and attention scores compared with sham (all p < 0.05), whereas differences in delayed recall did not reach significance (**Table 3**, **Figure 4B**).

GLM analysis demonstrated significant Time effects for all subdomains (all p < 0.001) and significant Time × Group interactions for FDS (F = 5.281, p = 0.025, partial η² = 0.073) and BDS (F = 14.827, p < 0.001, partial η² = 0.181), confirming that working-memory was more pronounced following iTBS treatment (**Table 2**).

### 3.4 Changes in serum biochemical markers

At baseline, serum concentrations of Hcy, CRP, and LDH were comparable between the two groups (all p > 0.05). After 4 weeks of intervention, both groups exhibited significant within-group reductions in these inflammatory biomarkers (all p < 0.05).

Notably, LDH levels decreased significantly more in the iTBS group than in the sham group (p < 0.05), while intergroup differences for Hcy and CRP were not statistically significant (**Table 3**, **Figure 4C**).

GLM results further supported a significant main effect of Time for all three markers (all p < 0.001), reflecting overall reduction after treatment, and a trend toward greater LDH reduction in the iTBS group (F = 2.899, p = 0.093, partial η² = 0.041; **Table 2**).

### 3.5 Correlations between cognitive and biochemical changes

To explore potential mechanistic relationships, Pearson correlation analyses were conducted between Δ scores in cognitive and biochemical measures (**Table 4**, **Figure 3**).

In the iTBS group, improvements in global and executive cognitive functions (ΔMMSE, ΔMoCA, ΔFAB, ΔFDS, ΔBDS) were significantly correlated with decreases in serum LDH (all r = –0.33 to –0.53, p < 0.05), while ΔFAB also correlated with reductions in Hcy (r = –0.486, p = 0.003). These associations suggest that cognitive gains were accompanied by biochemical modulation, particularly LDH reduction.

In contrast, in the sham group, ΔMMSE, ΔMoCA, ΔFDS, and ΔAttention were positively correlated with ΔHcy (r = 0.38–0.69, all p < 0.05), indicating that lesser Hcy reduction was associated with poorer cognitive improvement.

## 4 Discussion

### 4.1 Clinical significance and rationale

Early intervention in PSCI is crucial to prevent progression to vascular dementia or mixed-type Alzheimer’s disease. While rTMS has shown general benefits for cognitive deficits, evidence for iTBS in PSCI remains scarce. This study demonstrates that both groups experienced significant improvements in global cognition, with the iTBS group showing superior gains compared to sham stimulation. These findings suggest that iTBS may exert more potent neuromodulatory effects on cognitive recovery in PSCI, supporting its potential as an adjunctive non-pharmacological approach.

### 4.2 Neurophysiological mechanisms: left DLPFC, theta entrainment, and precision targeting

Our hypothesis—that restoring theta-frequency synchronization within the left DLPFC facilitates cognitive recovery—was supported by the present findings and aligns with emerging neurophysiological evidence. Consistent with previous meta-analyses, excitatory TMS targeting the left DLPFC has been shown to produce stronger improvements in global cognition, memory, attention, and executive control in PSCI patients compared with right-hemisphere or non-frontal stimulation sites (46). In line with this, recent randomized controlled trials reported that iTBS applied over the left DLPFC, when combined with structured cognitive training, enhanced global and executive functions and increased P300 amplitudes while shortening latency, suggesting improved neural efficiency and cognitive processing speed (47). A high-dose, individualized iTBS protocol guided by functional connectivity mapping of the fronto-cognitive network further yielded robust cognitive benefits without adverse effects (10).

Mechanistically, theta-patterned stimulation delivered intermittently is thought to entrain endogenous oscillations within the theta band (4–7 Hz) and promote long-term potentiation (LTP)-like plasticity in prefrontal–limbic circuits. This network-level modulation extends beyond local cortical excitability and may underlie the observed behavioral improvements in executive and working-memory domains. Functional near-infrared spectroscopy (fNIRS) and EEG studies have demonstrated that iTBS induces increased activation in frontopolar, orbitofrontal, and anterior cingulate regions, correlating with improvements on functional scales such as BI and LOTCA (48, 49). These findings underscore the distributed neuroplastic response of the DLPFC-centered control network following theta-burst entrainment.

Our study adds further evidence by showing that iTBS-induced cognitive gains were accompanied by significant reductions in serum lactate dehydrogenase (LDH) and homocysteine (Hcy), both of which have been linked to oxidative stress and neural injury. The significant negative correlations between ΔLDH and Δcognitive scores in the iTBS group suggest that cortical neuroplastic recovery may be associated with systemic metabolic normalization and anti-inflammatory modulation. This observation complements prior reports indicating that prefrontal stimulation enhances mitochondrial efficiency and downregulates peripheral stress markers (50, 51).

Taken together, these results support the notion that precision-targeted iTBS over the left DLPFC can recalibrate disrupted frontoparietal and limbic circuits through theta-band entrainment and metabolic modulation, leading to functional and biochemical recovery in PSCI patients. The clinically practical protocol adopted in this study (1200 pulses at 80% RMT over the left DLPFC) further demonstrates real-world feasibility for integrating neuromodulation into cognitive rehabilitation frameworks.

### 4.3 Domain-specific cognitive recovery: working memory, executive function, and everyday function

Our trial demonstrated that iTBS significantly enhanced working memory (FDS, BDS), executive processing (FAB), and activities of daily living (BI) more than sham stimulation. These findings are consistent with the growing literature demonstrating that iTBS targeted to the left DLPFC can selectively improve higher-order cognitive domains in PSCI and related populations (46, 52).

A recent three-arm RCT at Peking University directly compared high-dose (3600 pulses/day) iTBS, standard-dose (1200 pulses/day) iTBS, and sham controls in PSCI patients. Both active groups improved on MoCA, but the high-dose strategy produced significantly greater gains versus control and the standard-dose arm (10). Improvements extended across secondary outcomes including Wechsler Memory Scale and WAIS working-memory indices, showing a dose-dependent effect on memory and executive domains. Meanwhile, a network meta-analysis synthesizing data from 12 RCTs (n ≈ 506) concluded that iTBS and conventional rTMS both significantly improve global cognition and daily functioning in PSCI, but with stronger effect sizes for executive and working-memory components in iTBS-treated patients, although heterogeneous methodology limits conclusions (53, 54).

Moreover, an iTBS-plus-cognitive-training study in NeuroRehabilitation (2023–2024) using fNIRS demonstrated that combined treatment enhanced visuomotor organization and thinking operations (LOTCA domains), accompanied by activation changes in left DLPFC, prefrontal polar cortex and Broca’s region — brain areas implicated in executive and working-memory control (9). In parallel, a meta-analysis of post-stroke iTBS for upper limb motor recovery reported concomitant improvements in BI, reinforcing the link between enhanced executive-motor integration and daily function recovery (55). Lastly, broader reviews in stroke rehabilitation confirm that domain-focused cognitive interventions, whether behavioral or neurostimulatory, yield selective enhancements in memory and executive subdomains and daily functioning — with iTBS showing particular promise for working-memory transfer and functional independence (48).

Thus, our results align with and extend the existing body of evidence, indicating that left DLPFC-applied iTBS facilitates not only global cognition but selectively bolsters working memory and executive control, translating into meaningful gains in everyday function. These cognitive-domain level improvements support the mechanistic rationale of hemispheric excitation balance re-establishment and network-level synchronization integration in rehabilitation.

### 4.4 Anti-inflammatory and neuroprotective mechanisms of iTBS: biochemical modulation and cognitive coupling in PSCI

Our findings indicate that iTBS significantly reduced serum LDH levels compared with sham stimulation, while Hcy and CRP decreased in both groups without statistically significant intergroup differences. Importantly, correlation analyses revealed that reductions in LDH and Hcy were significantly associated with improvements in global cognition (ΔMMSE, ΔMoCA) and executive-working-memory performance (ΔFAB, ΔFDS, ΔBDS), suggesting a biochemical–behavioral coupling mechanism (**Table 4**, **Figure 3**).

A 2024 meta-analysis of repetitive TMS (rTMS) across various neurological and psychiatric disorders reported consistent improvements in peripheral inflammatory markers alongside cognitive gains, underscoring the potential of inflammation modulation to mediate clinical recovery (18, 56). In patients with PSCI, a prospective South Korean trial applying high-frequency rTMS over the ipsilesional DLPFC documented significant reductions in blood IL-6 and IL-1β immediately after treatment, which remained lower at three months and correlated strongly with improvements in verbal memory and visuospatial functioning (57).

Although cytokine studies have dominated the literature, large-scale cohort investigations demonstrate that elevated CRP and Hcy are independent predictors of early cognitive decline after stroke. For example, the Nor-COAST study (2023–2024) found that higher CRP-to-lymphocyte and globulin-to-lymphocyte ratios were associated with increased PSCI risk (4). Our results extend this evidence by showing that iTBS-induced reductions in LDH may reflect enhanced neuronal integrity and reduced oxidative injury. LDH serves as a metabolic stress marker reflecting both astrocytic glycolytic activity and tissue hypoxia; its decline may indicate improved cerebral energy metabolism and reduced cell-damage load following neuromodulation.

While LDH has been less extensively studied, its elevation is widely recognized as a marker of neuronal injury and tissue hypoxia, and reductions may reflect improved cellular integrity. Recent neurophysiological evidence shows that rTMS applied to cortical motor and prefrontal regions can modulate oxidative stress and decrease serum LDH activity, paralleling behavioral recovery (58). This aligns with our observation that greater LDH reductions corresponded to stronger cognitive improvements, implying that metabolic restoration is an integral component of the neuroplastic response. Animal data further confirm that low-frequency rTMS can suppress LDH release and improve memory and learning after ischemia-hypoxia (56, 59).

In summary, this study is among the first to quantitatively evaluate Hcy, CRP, and LDH as systemic biomarkers of iTBS treatment in PSCI. The pronounced LDH reduction and its strong correlation with cognitive recovery support the hypothesis that iTBS ameliorates neuronal injury and metabolic dysregulation through both central (neuroplasticity, theta entrainment) and systemic (anti-oxidative, anti-inflammatory) pathways. These findings provide mechanistic insight into how iTBS facilitates cognitive rehabilitation and identify LDH as a promising candidate biomarker for monitoring neuromodulation efficacy in PSCI.

## 5 Advantages, tolerance and limitations

The iTBS paradigm features shorter duration, focused rhythmic pulses and higher patient tolerability compared to conventional rTMS, as reflected by lower dropout rates and better subjective acceptability in this trial. Its theta-rhythm alignment may improve precision targeting and cortical entrainment efficiency. However, the study limitations include: lack (1) Although the original power analysis estimated 78 participants to achieve sufficient statistical power, only 72 were enrolled and 69 completed the study. This modest shortfall may have slightly reduced the power to detect smaller between-group effects. To address this limitation, the analysis was strengthened using a two-way mixed General Linear Model (GLM), which accounts for both within- and between-subject variance and enhances the robustness of inference; (2) of neuroimaging validation to objectively confirm cortical activation changes; (3) absence of individualized stimulation parameter optimization (e.g., target site, intensity, frequency); and (4) enrollment solely of general cognitive impairment without subtype stratification (e.g., visuospatial deficits or calculation impairment), and limited assessment tools.

## 6 Future directions

Future research should incorporate neuroimaging (e.g., EEG or fMRI) to map iTBS-induced network changes and confirm engagement in cognitive circuits. Stratified RCTs comparing customized stimulation parameters across cognitive subtypes would enhance precision rehabilitation. Additionally, larger and longer-term studies are needed to establish optimal frequency selection (e.g., comparing 2, 5, 6 Hz), dose-response relationships, and durability of effects.

## Data Availability

The original contributions presented in the study are included in the article/supplementary material. Further inquiries can be directed to the corresponding author/s.
